# A Novel Process for Oleacein Production from Olive Leaves Using Freeze Drying Methodology

**DOI:** 10.3390/foods14020313

**Published:** 2025-01-18

**Authors:** Christina Koutra, Efi Routsi, Panagiotis Stathopoulos, Eleftherios Kalpoutzakis, Marina Humbert, Olivier Maubert, Alexios-Leandros Skaltsounis

**Affiliations:** 1 Division of Pharmacognosy and Natural Products Chemistry, Department of Pharmacy, National and Kapodistrian University of Athens, 15771 Athens, Greece; ckoutral@pharm.uoa.gr (C.K.); efroutsi@pharm.uoa.gr (E.R.); elkalp@pharm.uoa.gr (E.K.); 2 ROBERTET SA, 37 Avenue Sidi Brahim, 06130 Grasse, France; marina.humbert@robertet.com (M.H.); olivier.maubert@robertet.com (O.M.)

**Keywords:** *Olea europaea*, drying methods, olive tree by-products, bioactive compounds, enzymatic biotransformation

## Abstract

The abundant yet underutilized olive leaves, a renewable by-product of olive cultivation, offer untapped potential for producing high-value bioactive compounds, notably oleacein. Existing extraction methods are often inefficient, yielding low quantities of oleacein due to enzymatic degradation of its precursor, oleuropein, during conventional processing and storage. This study aimed to overcome these limitations by exploring a novel methodology based on freeze-drying, to facilitate the in situ enzymatic biotransformation of oleuropein into oleacein directly within the plant matrix. Olive leaves were subjected to three drying methods—ambient air drying, microwave drying, and freeze-drying—and their phenolic profiles were analyzed. The findings demonstrated that freeze drying uniquely promotes the selective activation of β-glucosidase and esterase enzymes while simultaneously inhibiting oxidative enzymes, such as polyphenol oxidase and peroxidase, resulting in significantly enriched oleacein content. This process eliminates the need for extensive post-extraction transformations, providing a cost-effective, scalable, and sustainable approach to oleacein production. The proposed methodology aligns with circular economy principles and holds substantial potential for applications in pharmaceuticals, nutraceuticals, and functional food industries.

## 1. Introduction

The olive tree (*Olea europaea* L., Oleaceae) is one of the most significant agricultural crops in the Mediterranean basin, extensively cultivated for both olive oil and table olives, which are key components of the Mediterranean diet [1]. In recent years, interest in the by-products of olive cultivation has grown due to increasing awareness of their potential contributions to a sustainable and circular economy [2]. Notably, olive leaves (OLs), abundantly produced during pruning and harvesting, are rich in bioactive compounds, making them promising resources for valorization [3]. Among these compounds, oleuropein (OLE), a secoiridoid glucoside, is particularly noteworthy due to its extensive pharmacological activities [4,5,6,7] and its potential as a precursor for other valuable secoiridoids, including oleacein (OLEA) [8,9] and oleocanthal [10,11]. OLE can be converted by glucosidases and esterases to the corresponding oleoside aldehydes [12], while oxidative enzymes, such as polyphenol oxidase (PPO) and peroxidase (POD), promote the oxidation and degradation of OLE leading to the production of various secoiridoid derivatives [13].

OLEA, a bioactive compound found mainly in high-quality extra virgin olive oil [14], has attracted scientific and commercial interest due to its diverse bioactive properties, including anti-inflammatory [15], antibacterial [16], antioxidant [17], anti-tumor [18], and anti-atherosclerotic effects [19]. In medicine, OLEA has demonstrated anti-inflammatory properties by modulating nitric oxide and arachidonic acid-dependent inflammatory pathways, suggesting its utility in managing chronic inflammatory conditions. Additionally, OLEA has been shown to improve lipid and glucose metabolism, indicating potential benefits in addressing metabolic disorders. In the nutraceutical sector, OLEA’s antioxidant and anti-inflammatory effects support its inclusion in dietary supplements aimed at promoting cardiovascular health. Cosmetic applications are also promising, as OLEA’s properties can be harnessed in formulations designed to combat oxidative stress and inflammation, thereby contributing to skin health. Furthermore, OLEA’s antimicrobial activity suggests its potential as a natural preservative in food and cosmetic products. These diverse applications underscore the significance of OLEA-enriched extracts in advancing health and wellness across multiple industries. The OLEA compound does not only contribute to Extra Virgin Olive Oil (EVOO)’s health benefits but also enhances its sensory characteristics, particularly its bitterness, pungency, and astringency [20,21]. However, OLEA’s content in olive oil is limited and highly variable, influenced by many factors such as olive cultivar, agricultural practices, climatic conditions, soil composition, fruit maturity, and extraction parameters [22,23]. OLEA is produced through the transformation of OLE in olive fruits, a process involving enzymatic pathways, particularly the activation of methylesterases and β-glucosidases, which initiate OLE’s conversion upon the breakdown of the plant cell structure [24,25]. As a result, obtaining OLEA directly from olive oil requires substantial quantities, making the sourcing of OLEA at a gram scale challenging due to limited accessibility and high costs [26]. It is worth noting that the current cost of pure OLEA is very high [27].

Chemical synthesis is often an excellent route to obtain large amounts of a compound. The significant interest in OLEA has triggered the development of various synthetic approaches, including multistep total synthesis or one-step hemisynthesis from OLE. However, these methods typically result in low overall yields [28,29,30].

On the other hand, it has been observed that OLE levels in OLs decline significantly during long-term storage or exposure to humid environments. Specifically, the effects of temperature and relative humidity (RH) on OLE stability are well documented. Elevated temperatures and higher RH levels have been associated with accelerated OLE degradation, while low RH appears to offer a stabilizing effect, suggesting that controlled environmental conditions during storage play a crucial role in maintaining OLE content. Recent studies also indicate that OLE degradation leads to the formation of secondary metabolites, including hydroxytyrosol derivatives, which may alter the therapeutic profile of olive leaf extracts [31]. Additionally, the exposure of frozen olive leaf samples for a very short period to ambient temperatures has been shown to activate enzymatic processes, resulting in a rapid decline in OLE levels. This degradation appears to be driven by the breakdown of cell membranes, which, upon thawing, enables OLE to interact with endogenous enzymes that are located in different cellular compartments in fresh tissues. This compartmentalization, similar to that observed in olive fruits, likely preserves OLE content by restricting its conversion until the integrity of cell membranes is compromised [32]. Moreover, according to the literature, water extraction of OLs, particularly at low temperatures, can promote the conversion of OLE into its bioactive derivative, OLEA [33,34]. This transformation is facilitated by endogenous enzymes within the plant tissues, which become active in aqueous environments and catalyze the hydrolysis, deglycosylation, and decarboxylation of OLE, leading to the formation of OLEA. Such enzymatic activity is enhanced under mild extraction conditions that prevent thermal degradation, preserving and partially converting these valuable phenolic compounds. Notably, in 1996, Hansen et al. successfully isolated OLEA from an aqueous extract of OLs and demonstrated its bioactivity as an angiotensin-converting enzyme (ACE) inhibitor [33]. In a similar study on another species of the Oleaceae family (*Ligustrum vulgare*), aqueous extraction of fresh leaves led to the production of OLEA; however, the extraction yield was low [35].

Furthermore, the drying process of OLs significantly affects the phytochemical profile of the final extract [36]. Numerous drying methods, such as freeze drying (FD) [37], ambient air drying (AA) [38], and microwave drying (MW) [39], have been explored, which affect the stability and recovery of polyphenols in different ways. Freeze drying (FD), involving sublimation at low temperatures (−80 °C), is regarded as a mild method that preserves thermolabile compounds, while MW drying uses high-intensity radiation to rapidly dehydrate plant tissues. AA drying relies on capillary action and diffusion, leading to slower moisture removal but minimal cellular disruption [40]. Although some studies suggest that freeze-drying preserves OLE content, the impact of different drying methods remains understudied, with findings often contradictory [36,41]. Notably, variations in solvent extraction methods and drying conditions contribute to inconsistencies across studies, underscoring the need for systematic investigation.

The primary goal of our work was to investigate the levels of OLE in OLs, dried using three different methods (AA, MW, and FD), and to correlate the low OLE levels observed in OLs dried under ambient conditions or via freeze-drying with its potential biotransformation into other known secoiridoid derivatives, including OLEA. Specifically, this study explores the production of OLEA-enriched extracts from OLs by altering their drying conditions, as well as a secondary aim: the potential isolation of OLE, the most abundant and promising EVOO phenolic component, directly from OLs. By transforming OLs, an abundant agricultural by-product, into a source of highly valuable bioactive compounds, this approach aligns with circular economy principles, offering a scalable and economically viable pathway for producing bioactive compounds from plant-based materials.

## 2. Materials and Methods

### 2.1. Raw Materials and Chemical Reagents

The OLs used in this work originated from the University Campus of NKUA and were collected in May 2022 (sample codeː SP001). Standard compounds of OLE (purity ≥ 99%) were purchased from (Sigma-Aldrich Chemie GmbH, Steinheim, Germany), while OLEA (purity ≥ 99%) was obtained from (Pharmagnose SA, Athens, Greece). HPLC and LC grade methanol, Deuterium grade chloroform, formic acid, and orthophosphoric acid (purity ≥ 98%) were purchased from (Sigma Aldrich Chemie GmbH, Steinheim, Germany) and (Macron Fine Chemicals, Waltham, MA, USA), respectively. In different stages of the experimental procedure, analytical-grade methanol and ethyl acetate purchased from (Sigma Aldrich Chemie GmbH, Steinheim, Germany) was used.

### 2.2. Sample Preparation

Fresh OLs (20 kg) were divided equally into three batches. The first batch was dried under AA conditions, where the fresh leaves were spread on a tray lined with filter paper. The tray was left for 10 days in a shady and well-ventilated place. The second batch was frozen at −80 °C for 24 h and dried under vacuum (FD) using a Gellert Freeze Dryer Cryo Dryer 5 (Langweid am Lech, Germany) (−40 °C, 0.6 mbar). The third batch was dried using MW (MW, Milestone Inc. Srl, Sorisole—Italy) irradiation at 700 W for 1 h. After drying, all samples were pulverized using a cutting mill. The olive leaf powder was then sieved to obtain particles smaller than 0.71 mm and stored at 8–10 °C prior to extraction. The extraction of OLs was performed with methanol at 60 °C under stirring. The ratio of plant material mass to solvent volume was 1:6 (4 kg of dried OLs to 24 L of analytical-grade MeOH), with an extraction time of 1 h. The extracts were filtered under vacuum, evaporated to dryness using a rotary evaporator under reduced pressure, and then subjected to HPLC-DAD analysis.

### 2.3. HPLC-DAD Analysis

A high-performance liquid chromatography method combined with diode array detection (HPLC–DAD) was developed to determine the main phenolic compounds in the methanolic extracts of dried OLs. Determination of OLE and OLEA in OLs extracts was performed on a HPLC system (Thermo Scientific, Mississauga, ON, Canada) equipped with a SpectraSystem 1000 degasser, a SpectraSystem P4000 pump, a SpectraSystem AS3000 autosampler, and a UV SpectraSystem UV8000 Photo Diode Array (PDA) detector, by applying the IOC-proposed analytical method with some modifications. The IOC method was performed according to analytical conditions referred to in the IOC/T.20/Doc No. 29 method (International Olive Council, 2009) [42], and they have been described in our previous work [43]. Specifically, the separation of the components of the extracts was achieved on a reversed-phase Spherisorb Discovery HS C18 column (250 mm × 4.6 mm, 5 µm; Supelco, Bellefonte, PA, USA) using a mobile phase consisting of 0.2% aqueous orthophosphoric acid (A) and Methanol/Acetonitrile (50:50 *v*/*v*) (B), at a flow rate of 1.0 mL/min and under ambient temperature. The gradient elution was as follows: 0 min, 96% A and 4% B; 0–40 min, 96–50% A and 4–50% B; 40–45 min, 50–40% A and 50–60% B; 45–60 min, 40–0% A and 60–100% B; 60–70 min, 0% A and 100% B; 70–72 min, 0–96% A and 100–4% B; 72–82 min, 96% A and 4% B. All samples were diluted in 500 µL MeOH_HPLC grade_ and 500 µL H_2_O_HPLC grade_. The injection volume was held constant at 20 µL, and chromatograms were monitored at 280 nm. All analyses were made in triplicate. The determination of the main phenolic compounds in OLs extracts was achieved using the regression analysis method. Specifically, standard calibration curves for OLE and OLEA were prepared. For the OLE quantification, 8-point calibration curves were constructed (y = 22974x + 157567, r2 = 0.9980), while OLEA was quantified according to a 10-point calibration curve, respectively (y = 9063.6x + 7942.2, r2 = 0.9997). To calculate the Limit of Quantification (LOQ) for OLEA and OLE compounds based on their calibration curves y = ax + b the formula LOQ = 10(SD/α) was used, where sd is the standard deviation of the y-intercepts of multiple calibration curves, and *a* is the average slope of the calibration curves. This formula ensures that the LOQ corresponds to the lowest concentration of the compound that can be quantified with acceptable precision and accuracy, typically defined as a signal-to-noise ratio of 10. Data acquisition was monitored by the ChromQuestTM4.2 software (ThermoScientificTM, Mississauga, ON, Canada).

### 2.4. LC-ESI/LTQ-Orbitrap/MS/MS Analysis

The determination of OLE and OLEA in dried OLs was confirmed by further analyzing the methanolic extracts using a high-resolution mass spectrometer (HRMS) with orbitrap analyzer technology. For the UPLC-HRMS analysis, a Waters H-Class Acquity UPLC system (Waters, Milford, MA, USA) coupled to a Velos Pro-Orbitrap Elite hybrid mass spectrometer (ThermoScientific, Waltham, MA, USA) was utilized. Separation was performed on a Supelco (Supelco, Bellefonte, PA, USA) Ascentis Express C-18 reverse phase column (150 mm × 2.1 mm, 2.0 µm) at a stable temperature of 40 °C. Measurements were performed with a total acquisition time of 20 min and a flow rate of 300 µL/min. The injection volume was 10 µL, and the autosampler temperature was set at 10 °C. The mobile phase was a 0.1% formic acid aqueous solution (A) and acetonitrile (B), and the following gradient elution was applied for chromatographic separation of analytes: 0 min, 95% A and 5% B; 1–15 min, 95–0% A and 5–100% B; 15–17 min, 0% A and 100% B; 17–17.5 min, 0–95% A and 100–5% B; 17.5–20 min, 95% A and 5% B. Mass spectra were obtained in negative ionization mode using a heated electrospray (HESI) source. The HESI conditions were as follows: capillary and heater temperature were set at 350 °C; source voltage was set at 2.7 kV. Sheath and auxiliary gas were adjusted to 45 and 15 arbitrary units, respectively. The HRMS data were acquired in full scan mode in the range of 113–1000 *m*/*z*, with a resolving power of 60,000 at 500 *m*/*z* and a scan rate of 1 microscan per second. HRMS/MS experiments were obtained in data-depending method with a collision energy of 35.0% (q = 0.25).

The raw data were acquired and processed with Thermo Xcalibur Version 2.2. Spectrometric features, such as accurate *m*/*z*, proposed elemental composition (EC), observed isotopic patterns, and ring double bond equivalent (RDBeq) values, were used to identify molecular ions and correctly assign elemental formulas. The HRMS/MS experiments, in combination with existing in-house databases, significantly contributed to the determination of the structures of the components of each extract. The data were recorded at the end of the process and compared with the literature data.

### 2.5. Isolation of OLEA from the Methanolic Extract of FD-Dried OLs

#### 2.5.1. Liquid–Liquid Extraction

A total of 1.35 Kg of FD-dried OLs’ methanolic extract, prepared as described in Section 2.2, was diluted in 3 L of distilled water. Afterwards the resulting aqueous extract was subjected to sequential liquid–liquid extraction with 3 L of ethyl acetate (EtOAc), repeated three times to ensure exhaustive extraction. The combined EtOAc extract was then evaporated to dryness using a rotary evaporator under reduced pressure, yielding 0.46 kg of EtOAc extract.

#### 2.5.2. Preparative High-Performance Liquid Chromatography (Prep—HPLC)

The isolation of OLEA from the EtOAc extract of FD-dried OLs (produced as described in Section 2.5.1) was performed on an ECOM preparative HPLC system (Chrášťany u Prahy, Czech Republic) equipped with two pumps, a rotary injection valve with a loop compartment of 5 mL capacity, and an ECOM TOY18DAD photodiode array detector. The separation was achieved on a reversed-phase column (C18, 50 mm × 50 mm, 10 µm) using a mobile phase that consisted of water (A) and methanol–acetonitrile (50:50) (B) with a flow rate of 30 mL/min, according to the International Olive Council (IOC) method [42]. The gradient elution was as follows: 0 min, 96% A and 4% B; 40 min, 50% A and 50% B; 45 min, 40% A and 60% B; 50 min, 100% B; 55 min, 100% B; 58 min 96% A and 4% B; 60 min 96% A and 4% B. All samples were prepared by diluting 1 g of extract in 2.5 mL MeOH_HPLC grade_ and 2.5 mL H_2_O_HPLC grade_ and were filtered prior to injection. Detection was carried out at 280 nm, and the data processing was performed with ClarityTM 8.0 software. The peak corresponding to OLEA was collected manually and evaporated to dryness, resulting in highly pure OLEA (purity > 95%).

### 2.6. Nuclear Magnetic Resonance Spectroscopy (NMR)

The structural elucidation of OLEA was performed using a Nuclear Magnetic Resonance Spectroscopy (NMR) Advance III Bruker BioSpin device with a 5 mm BBI probe at 600 MHz (Karlsruhe, Germany). The dilution solvent of analytes was deuterium-grade chloroform (CDCl_3_). The chemical shifts were expressed as δ (ppm), and the coupling constants J were expressed as Hertz (Hz). The multiplicity of the spectral peaks was expressed as s (singlet), brs (broad singlet), d (doublet), t (triplet), q (quartet), dd (double of doublets), and m (multiplet).

### 2.7. Statistical Analysis

A pairwise comparison of the mean values of samples dried with three different drying methods was carried out via a two tailed *t*-test at *p* < 0.05. The analyses were carried out with SPSS 14.0 software (SPSS Inc., Chicago, IL, USA).

**Figure 1:**
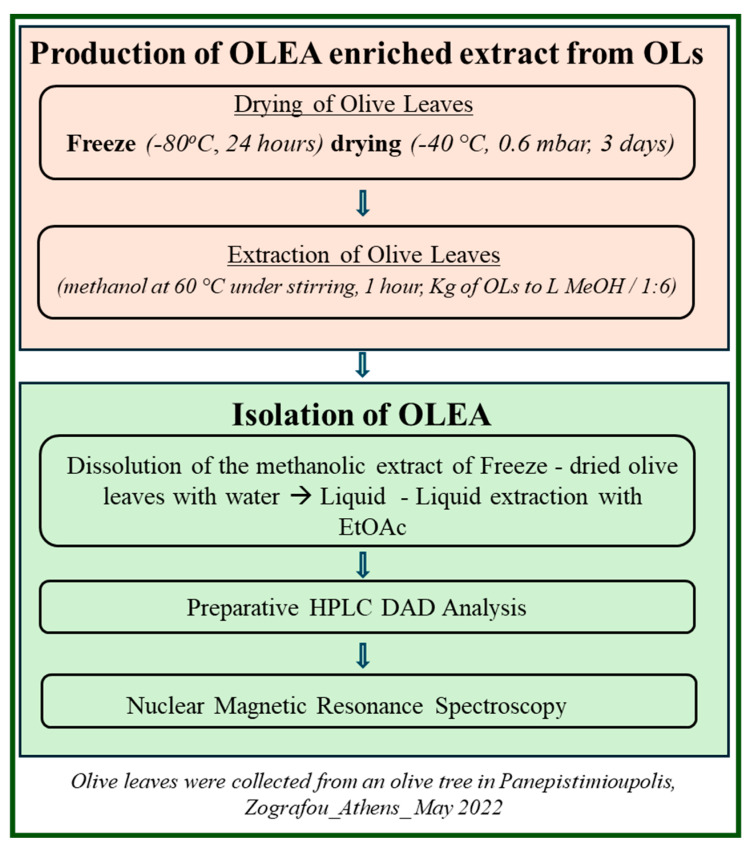
Schematic representation of the experimental procedures.

Figure 1 provides an overview of the experimental procedures undertaken to produce OLEA-enriched extracts from OLs and to isolate OLEA, a highly valued bioactive secoiridoid of extra virgin olive oil (EVOO), with high purity.

## 3. Results and Discussion

### 3.1. Exploring OLE and OLEA Content in AA-, MW-, and FD-Dried OLs

The first step of this study was to examine the impact of drying methods in determining the quality, composition, and extractability of bioactive compounds from OLs, which are known for their rich polyphenolic content.

The total water content of the OLs prior to drying was measured at 38.39%. Both FD and MW drying effectively reduced the water content to levels below the European Pharmacopoeia requirements of 10% for olive leaves, with MW demonstrating slightly greater efficiency. Specifically, the moisture content (MC) remaining after MW was 4.76%, while FD resulted in a MC of 5.13%. The AA drying method was slightly less effective, yielding a MC of 5.27%.

These findings are in agreement with the literature and are better understood when considering the distinct dehydration mechanisms associated with each drying technique [44]. In MW drying, high-intensity irradiation is absorbed by the intrinsic water content of the plant material, increasing the kinetic energy of water molecules and generating high-energy steam within the cellular structure. This process results in the disruption of cellular compartments and rapid dehydration of the plant material. Notably, the temperature during MW drying is sufficiently controlled to prevent damage to critical quality parameters. The short duration of the MW drying process, combined with minimal energy losses due to the direct absorption of MW energy by the intrinsic water without the need for a medium, makes this technique the most effective drying method [44].

The freeze-drying process operates through the sublimation of ice crystals formed within plant cells during an initial freezing phase, typically conducted at approximately −80 °C. Achieving a supercooled state—wherein the material’s temperature is reduced below its cryoscopic threshold—is advantageous, as it promotes homogeneous ice nucleation throughout the entire volume, thereby accelerating the freezing process. This freezing stage is critical for minimizing chemical, biochemical, and microbiological transformations within the material, while simultaneously establishing a specific ice crystal structure. The ice crystal structure directly affects the subsequent sublimation phase by either facilitating or impeding water vapor migration, thereby influencing the rate of mass transfer and shaping the final morphology of the dried product [45]. Under conditions of low temperature and high vacuum, the removal of ice crystals via sublimation leads to cell lysis. The FD process, characterized by moderate temperature exposure and an anaerobic environment, effectively preserves thermolabile compounds, making it a mild and effective dehydration method that maintains the integrity of sensitive bioactive constituents [46].

Conversely, AA drying initiates dehydration through the evaporation of surface moisture, followed by capillary action and diffusion mechanisms to remove intrinsic water from within the plant tissue. This process induces cell shrinkage rather than lysis, resulting in a less efficient drying process [47]. Consequently, AA requires an extended duration—approximately 10 days—to achieve complete dehydration, compared to the 3 days typically required for FD and the mere 1 h necessary for MW drying.

After drying, all OLs samples were subjected to a standardized extraction protocol to evaluate the effects of the drying methods on extractability while controlling variables such as extraction solvent and temperature. The results showed that MW-dried OLs yielded the highest amount of extract, specifically 1.37 kg of dry extract per 4 kg of dried OLs. The extraction yield of FD-dried OLs was slightly lower, at 1.35 kg of dry extract/4 kg of dried OLs, while AA-dried OLs exhibited the lowest yield, at 1.04 kg of dry extract per 4 kg of dried OLs. Table 1 summarizes these results, expressed in % *w*/*w* values.

Regarding the extraction efficiency of the plant material post-drying, MW emerges as the most effective method, closely competing with FD. The substantial reduction in moisture content achieved through both MW and FD facilitates the pulverization of the plant material, resulting in a homogeneous particle size that enhances the surface area contact between the plant material and the extraction solvent. Moreover, the destruction of cellular compartments during these drying processes promotes the release of polyphenols into the extraction solvent, thereby increasing extraction yields. In contrast, AA drying is less effective, as the intact structure of the robust plant cell walls hinders polyphenols extraction compared to the FD and MW techniques.

In the next step, the HPLC DAD analysis of the methanol extracts of OLs dried in three different ways showed that, when using AA-dried OLs extracted with methanol as a reference protocol, OLE was quantified at 20.70% *w*/*w* in the extract (corresponding to 215 g of OLE in the final AA-dried OLs methanolic extract). In comparison, methanolic extracts from MW-dried OLs exhibited a significant increase in OLE yield, reaching 35.39% *w*/*w* in the extract, or 485 g of OLE in the final MW-dried OLs methanolic extract.

Table 1 summarizes the total extraction yield and the quantitative determination of OLE and OLEA in OLs processed using three different drying methods, along with their respective methanolic extracts.

**Figure 2:**
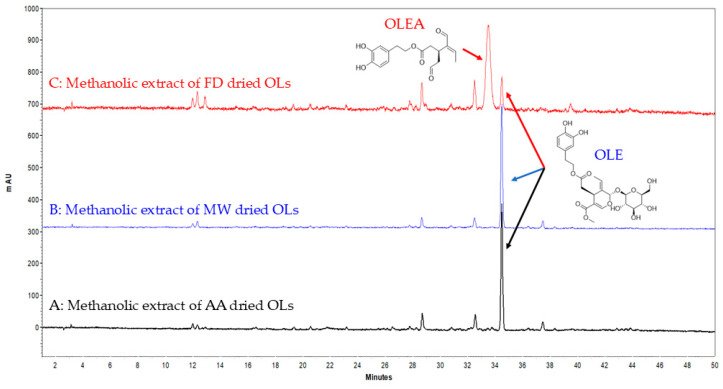
HPLC DAD chromatograms of the methanolic extracts of MW-, FD-, AA-dried OLs. The highlighted peaks are representative of OLE and OLEA.

Surprisingly, extracts derived from FD-dried OLs exhibited a significantly lower OLE content, measured at less than 2.5% *w*/*w* (Table 1). Additionally, an unexpected peak was observed near the OLE retention time ([rt] 34.6 min), appearing at [rt] 33.8 min. Analysis of the UV spectrum of this peak (λmax = 225 nm, 280 nm) indicated structural similarities to OLEA. Given our established expertise in OLEA within the laboratory [30] and by comparing the retention time and the UV spectrum of the unidentified peak with those of the secoiridoids’ analytical standards, it was confirmed that the peak eluting at 33.8 min corresponds to OLEA. (Figure S1, Supplementary Materials). Quantitative analysis revealed that extracts from FD-dried OLs were enriched in OLEA, with a concentration of 14.81% *w*/*w* (Figure 2).

To confirm the results of the HPLC-DAD analyses, the samples were further analyzed using an ultra-high-performance liquid chromatography system (UHPLC) coupled with a quadrupole Orbitrap high-resolution mass spectrometer (Q-Orbitrap HRMS). The LC-HRMS data are given as Supporting Information and were compared with HPLC- DAD findings. According to LC-HRMS analysis, in the methanolic extracts of AA-dried OLs (Figure S2, Supplementary Materials), the main chromatographic peak eluting at [rt] = 6.66 min corresponds to a mass spectrum in which the major pseudomolecular ion [M-H]- is presented at *m*/*z* 539.1761 Da. The proposed elemental composition of [M-H]- is C_25_H_31_O_13_ with a mass error of 0.1396 ppm, an RDBeq. of 10.5 in negative ion mode, and HRMS/MS major fragment ions at *m*/*z* 377.1242, 307.0824, and 275.0927, indicating that this ion at *m*/*z* 539.1761 Da corresponds to OLE. Additionally, in this LC-HRMS chromatogram, the OLEA compound was not detected. Similar results were obtained after the analysis of the methanolic extract of MW dried OLs (Figure S3, Supplementary Materials), where the mass spectrometric features of the main chromatographic peak, eluting at [rt] = 6.67 min, perfectly matched those of OLE as well ([M-H]^−^ at *m*/*z* 539.1754; EC: C_25_H_31_O_13_, RDBeq. 10.5 in negative ion mode, and HRMS/MS major fragment ions at *m*/*z* 377.1244, 307.0826, and 275.0928). Τhe pseudomolecular ion [M-H]^−^ of OLEA was also not detected. In contrast to the AA- and MW-dried OLs, the LC-HRMS chromatographic profile of the methanolic extract of FD-dried OLs presented significant differences. Specifically, in the LC-HRMS chromatogram of the FD-dried OLs (Figure S4, Supplementary Materials), apart from OLE, an additional peak appeared at a retention time [rt] = 7.12 min, where its spectrometric data ([M-H]^−^ at *m*/*z* = 319.1186; ECː C_17_H_19_O_6_; RDBeq. 8.5 in negative ion mode, and MS/MS fragmentation pattern at *m*/*z* 195.0662, 165.0556, and 301.1075) perfectly matched those of OLEA. These observations are in agreement with the results of the HPLC-DAD analyses described above. At this point, it is worth noting that the intensities of the pseudomolecular ions of OLE and OLEA in the LC-HRMS chromatogram are not quantitatively related to their content in the methanolic extract, as these compounds ionize to different degrees. Thus, the low signal intensity observed for OLEA can be attributed to its weaker ionization efficiency compared to OLE, which exhibits better ionization under the same HRMS conditions.

### 3.2. Proposed Biotransformation of OLE into OLEA in FD-Dried OLs

The different levels of OLE in the methanolic extracts of AA-, MW-, and FD-dried OLs, as well as the presence of OLEA in the methanolic extract of FD-dried OLs, can be explained by considering the following observations.

**Figure 3:**
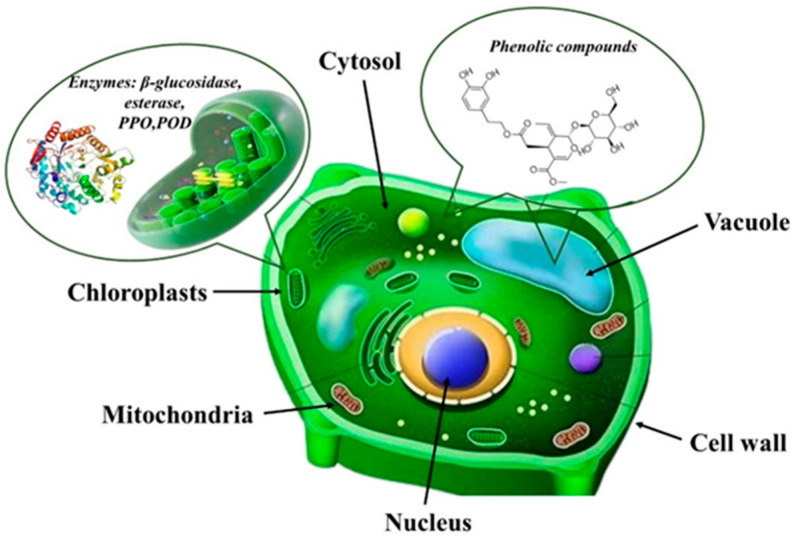
Polyphenols and endogenous enzymes’ location into the plant cell.

According to existing literature, endogenous enzymes (β-glucosidase, esterase, PPO, and POD) in plant cells of Oleaceae species (e.g., *Ligustrum obstifolium*) are primarily localized within chloroplasts, while polyphenolic compounds, including OLE, are predominantly distributed in the cytosol and vacuoles (Figure 3) [48]. Additionally, Sa et al., 2008, in their work, correlate the levels of OLE concentrations with the drying method of OLs, pointing out that enzyme–polyphenol interactions affect the stability and content of OLE in dried OLs [32].

In this work, based on the results of HPLC-DAD analyses, the highest levels of OLE were observed in the methanolic extract of MW-dried OLs. This finding aligns with the results of Latorre et al., 2012 and is probably associated with MW radiation, which inactivates enzymes, maximizing the OLE content in the dried OLs [49]. Specifically, high-intensity MW radiation inactivates the endogenous enzymes (β-glucosidase, esterase, PPO, and POD) in OLs, preventing enzymatic reactions related to the biotransformation, hydrolysis, and degradation of OLE [49]. Thus, during MW drying, although the plant material’s cell walls are disrupted, the enzymes have lost their activity, leaving the OLE molecule unaffected. This explains why OLs dried using MW treatment exhibit the highest OLE content compared to other drying methods, supporting the hypothesis that enzyme inactivation is essential for maintaining higher OLE levels.

In the case of naturally dried OLs, the OLE content was lower compared to MW-dried OLs. This is probably due to the fact that during natural drying the enzymes are not inactivated, as occurs in MW-assisted drying, resulting in partial degradation of OLE. Specifically, during AA drying, the gradual dehydration of the leaves causes cell shrinkage rather than cell lysis. Thus, the endogenous enzymes in OLs remain active for an extended period, favoring enzymatic reactions such as the biotransformation, hydrolysis, and degradation of OLE, which results in its content in the leaves being significantly reduced. These findings are consistent with studies by Sa et al. (2008) and Sahin et al. (2018), which demonstrate that high-humidity storage (75% relative humidity) and elevated temperatures accelerate OLE degradation following first-order kinetics, while lower humidity levels help stabilize OLE content [32,38].

Additionally, many studies report a dramatic reduction in OLE levels after freezing and then thawing OLs at ambient temperature [47]. This is probably due to the fact that during the freezing–thawing process, the cell walls of the plant material are ruptured, allowing enzymes and polyphenols, which physiologically under natural cell function conditions are located in different cellular compartments, to interact with each other. This interaction promotes enzymatic oxidation and degradation reactions of OLE, primarily driven by the activity of the oxidative enzymes PPO and POD.

**Figure 4:**
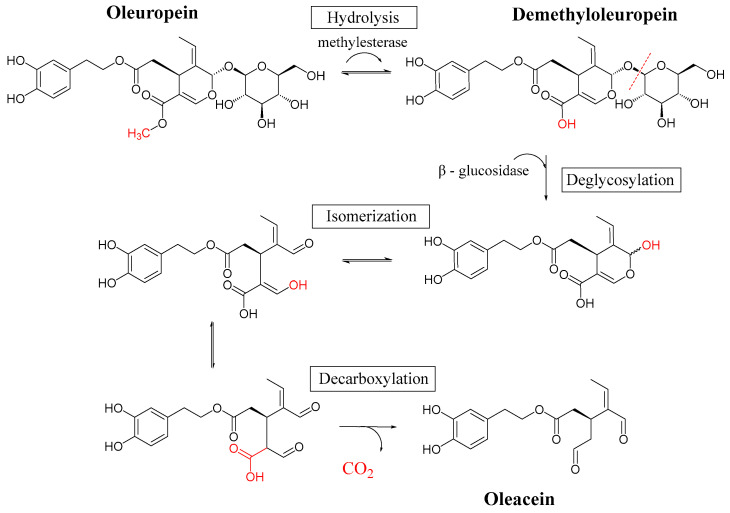
Proposed mechanism of biotransformation of OLE to OLEA.

However, during FD, the activity of PPO and POD is inhibited by anaerobic conditions, while β-glucosidase and esterase remain active, catalyzing the biotransformation of OLE into OLEA. Specifically, esterase facilitates the demethylation of OLE’s methyl ester, while β-glucosidase catalyzes the deglucosylation of its sugar unit. These modifications to the OLE iridoid ring led to the formation of the dialdehydic form of OLEA (Figure 4). This probably explains the presence of OLEA in FD-dried OLs.

An especially interesting aspect of this study is that the proposed biotransformation of OLE into OLEA, under FD conditions, occurs within the plant material itself, rather than during the extraction of OLs, using a specific extraction protocol. To our knowledge, this has not been previously reported in the literature. There are many scientific papers that describe the enzymatic transformation of OLE and the OLEA-enriched extracts’ production from OLs and leaves of other Oleaceae species (e.g., *Olea lancea* [33], *Ligustrum vulgare* [35,50], and *Ligustrum obstifolium* [48]), but these studies refer to aqueous extracts of OLs under specific extraction conditions. In these cases, the yields of OLEA-enriched extracts are quite low because water is an ineffective solvent for recovering polyphenolic components from OLs. The advantage of the proposed methodology for OLEA production from olive leaves is that this process for the treatment of FD-dried OLs can be performed with more efficient extraction solvents, such as methanol, which facilitate the recovery of polyphenolic components, including OLEA, from the plant material. This approach results in the production of high-yield OLEA-extracts. 

### 3.3. Isolation of OLEA from FD-Dried OLs

**Figure 5:**
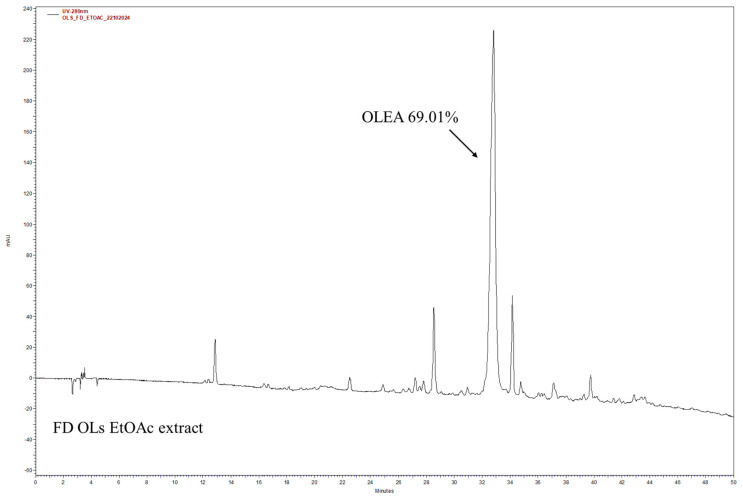
HPLC-DAD chromatogram of the EtOAc. The highlighted peak represents OLEA (rt = 33.8 min).

The next step of this work was to develop a simple, rapid, and cost-effective methodology for the pilot-scale production of OLEA-rich extracts and the isolation of high- purity OLEA. For this purpose, the FD-dried olive leaf methanolic extract, prepared as described in Section 2.2, was diluted in distilled water and extracted three consecutive times with ethyl acetate. The yield of the above process was 34.07%, and the purity of OLEA was 69.01%. (Figure 5).

**Figure 6:**
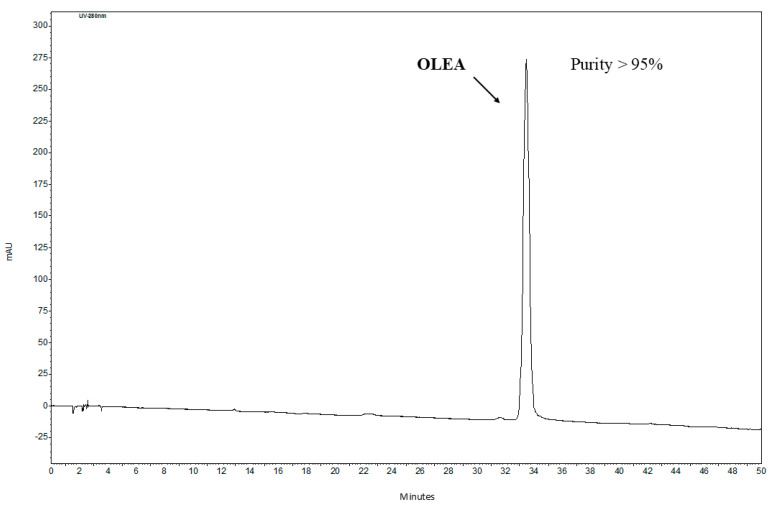
HPLC-DAD chromatogram of the isolated OLEA compound.

Further purification of OLEA was performed using a preparative chromatographic system (prep-HPLC-DAD) under the conditions described above, resulting in high-purity OLEA (HPLC purity ≥ 95%, Figure 6). The preparative HPLC-DAD chromatogram of the EtOAc extract is provided in the Supplementary Data (Figure S5, Supplementary Materials).

The structural elucidation was carried out using nuclear magnetic resonance (NMR) spectroscopy. The NMR data are presented in the Supplementary Data (Figures S6–S9).

## 4. Conclusions

This study presents a novel and efficient methodology for the production of OLEA, a high-value bioactive secoiridoid of EVOO, directly from OLs using a freeze-drying approach. The proposed methodology is based on the biotransformation of OLE into OLEA, which occurs under freeze-drying (FD) conditions within the plant material itself, rather than during the extraction process of OLs using a specific extraction protocol. The results of this study confirm that freeze-drying facilitates the selective activation of key enzymes, such as β-glucosidase and esterase, while simultaneously deactivating oxidative enzymes, including polyphenol oxidase and peroxidase. The advantage of the proposed methodology for OLEA production from OLs is that the treatment of FD-dried OLs can be performed with more efficient extraction solvents, such as methanol, which facilitate the recovery of polyphenolic components, including OLEA, from the plant material. This approach results in the production of high-yield OLEA extracts. By transforming OLs, an abundant agricultural by-product, into a source of highly valuable bioactive compounds, this approach aligns with the principles of the circular economy and sustainable resource management. The process offers a dual benefit: reducing agricultural waste while generating high-value, OLEA-enriched extracts with potential applications in pharmaceuticals, nutraceuticals, and functional foods.

## 5. Patents

The authors Christina Koutra, Panagiotis Stathopoulos, Marina Humbert, Olivier Maubert, and Alexios—Leandros Skaltsounis were coauthors in a patent relative to this work-titled as “METHOD FOR OBTAINING AN ENRICHED OLEACEIN EXTRACT FROM OLIVE LEAVES”-Publication Date: 2024-01-03, Application Number: EP22181324.9, Filing Date: 2022-06-27, IPC: A61K36/63, Patent Number: EP4299068A1-.

## Figures and Tables

**Table 1:** Extraction yield of methanol extracts of OLs dried with three different methods and quantitative determination of the target compounds OLE/OLEA in dry OLs and the respective methanol extracts.

Drying Method	AA *	FD *	MW *
Yield of extraction, (g dry extract/ Kg dry OLs)	260.90 ± 8.32	338.00 ± 7.54	343.00 ± 8.57
g OLE/100 g methanolic extract of OLs	20.70 ± 1.24	2.14 ± 0.76	35.39 ± 3.89
g OLEA/100 g methanolic extract of OLs	<LOQ ^1^	14.81 ± 3.77	<LOQ ^1^
g OLE/Kg dry OLs	54.01 ± 4.38	7.23 ± 2.61	121.40 ± 6.39
g OLEA/Kg dry OLs	<LOQ ^1^	50.10 ± 3.14	<LOQ ^1^

^1^ LOQ: Limit of Quantification (LOQ_OLE_ =10.00(SD/22974), LOQ_OLEA_ =10.00(SD/9063.6)). * Values are means of samplings analyzed in triplicate (*n* = 3) ± SD (SD: standard deviation).

## Data Availability

The original contributions presented in this study are included in this article/Supplementary Material; further inquiries can be directed to the corresponding authors.

## Supplementary Materials

The following supporting information can be downloaded at https://www.mdpi.com/article/10.3390/foods14020313/s1: Figure S1. HPLC-DAD chromatograms (at 280 nm) of the methanolic extracts of FD-dried OLs overlayed with the standard compounds of OLE and OLEA. The highlighted peaks are representative of OLE and OLEA. Figure S2. Base peak chromatogram obtained by LC-ESI/LTQ-Orbitrap/MS analysis of the methanolic extract of AA-dried OLs (A), typical extracted ion chromatogram of OLE at *m*/*z* 539.1763 (B), and typical extracted ion chromatogram of OLEA at *m*/*z* 319.1186 (C). Figure S3. Base peak chromatogram obtained by LC-ESI/LTQ-Orbitrap/MS analysis of the methanolic extract of MW-dried OLs (A), typical extracted ion chromatogram of OLE at *m*/*z* 539.1754 (B), and typical extracted ion of OLEA at *m*/*z* 319.1186 (C). Figure S4. Base peak chromatogram obtained by LC-ESI/LTQ-Orbitrap/MS analysis of the methanolic extract of FD-dried OLs (A), typical extracted ion chromatogram of OLE at *m*/*z* 539.1763 (B), and typical extracted ion chromatogram of OLEA at *m*/*z* 319.1186 (C). Figure S5. Preparative HPLC-DAD chromatogram after liquid–liquid extraction with EtOAc of the methanolic extract of FD-dried OLs, with the OLEA peak eluting at rt = 34 min highlighted. Figure S6. 1H NMR spectrum of oleacein in CDCl_3_ (600 MHz). Figure S7. 13C NMR spectrum of oleacein in CDCl_3_ (151 MHz). Figure S8. HSQC spectrum of oleacein in CDCl_3_ (600 MHz). Figure S9. HMBC spectrum of oleacein in CDCl_3_ (600 MHz).
