# Supplementary material for: A Novel Process for Oleacein Production from Olive Leaves Using Freeze Drying Methodology
Source: Foods. 2025 Jan 18;14(2):313. doi: 10.3390/foods14020313 (PMC11764853; doi:10.3390/foods14020313)
Supplement: Supplementary file 1 [file foods-14-00313-s001.zip › foods-3392547-supplementary.pdf]

Supplementary data

A Novel Process for Oleacein Production from Olive Leaves Using Freeze Drying Methodology

Christina Koutra [1], Efi Routsi [1], Panagiotis Stathopoulos [1,*], Eleftherios Kalpoutzakis [1], Marina Humbert [2], Olivier Maubert [2] and Alexios-Leandros Skaltsounis [1,*]

[1] Division of Pharmacognosy and Natural Products Chemistry, Department of Pharmacy, National and Kapodistrian University of Athens, 15771 Athens, Greece; ckoutral@pharm.uoa.gr (C.K.); efroutsi@pharm.uoa.gr (E.R.); elkalp@pharm.uoa.gr (E.K.)
[2] ROBERTET SA, 37 Avenue Sidi Brahim, 06130 Grasse, France; marina.humbert@robertet.com (M.H.); olivier.maubert@robertet.com (O.M.)
* Correspondence: stathopan@pharm.uoa.gr (P.S.); skaltsounis@pharm.uoa.gr (A.-L.S.)

Supplementary data index

Figure S1. HPLC-DAD chromatograms (at 280 nm) of the methanolic extracts of FD OLs overlayed with the standard compounds of OLE and OLEA. The highlighted peaks are representative of OLE and OLEA.

Figure S2. Base Peak Chromatogram obtained by LC-ESI/LTQ-Orbitrap/MS Analysis of the methanolic extract of AA dried OLs (A), Typical Extracted Ion Chromatogram of OLE at m/z 539.1763 (B), Typical Extracted Ion Chromatogram of OLEA at m/z 319.1186 (C)

Figure S3. Base Peak Chromatogram obtained by LC-ESI/LTQ-Orbitrap/MS Analysis of the methanolic extract of MW dried OLs (A), Typical Extracted Ion Chromatogram of OLE at m/z 539.1754 (B), Typical Extracted Ion of OLEA at m/z 319.1186 (C)

Figure S4. Base Peak Chromatogram obtained by LC-ESI/LTQ-Orbitrap/MS Analysis of the methanolic extract of FD dried OLs (A), Typical Extracted Ion Chromatogram of OLE at m/z 539.1763 (B), Typical Extracted Ion Chromatogram of OLEA at m/z 319.1186 (C)

Figure S5. Preparative HPLC-DAD Chromatogram after liquid-liquid extraction with EtOAc of the methanolic extract of FD OLs, with the OLEA peak eluting at rt = 34 minutes highlighted.

Figure S6. ^{1}H NMR spectrum of oleacein in $CDCl_3$ (600 MHz).

Figure S7. ^{13}C NMR spectrum of oleacein in $CDCl_3$ (151 MHz).

Figure S8. HSQC spectrum of oleacein in $CDCl_3$ (600 MHz).

Figure S9. HMBC spectrum of oleacein in $CDCl_3$ (600 MHz).

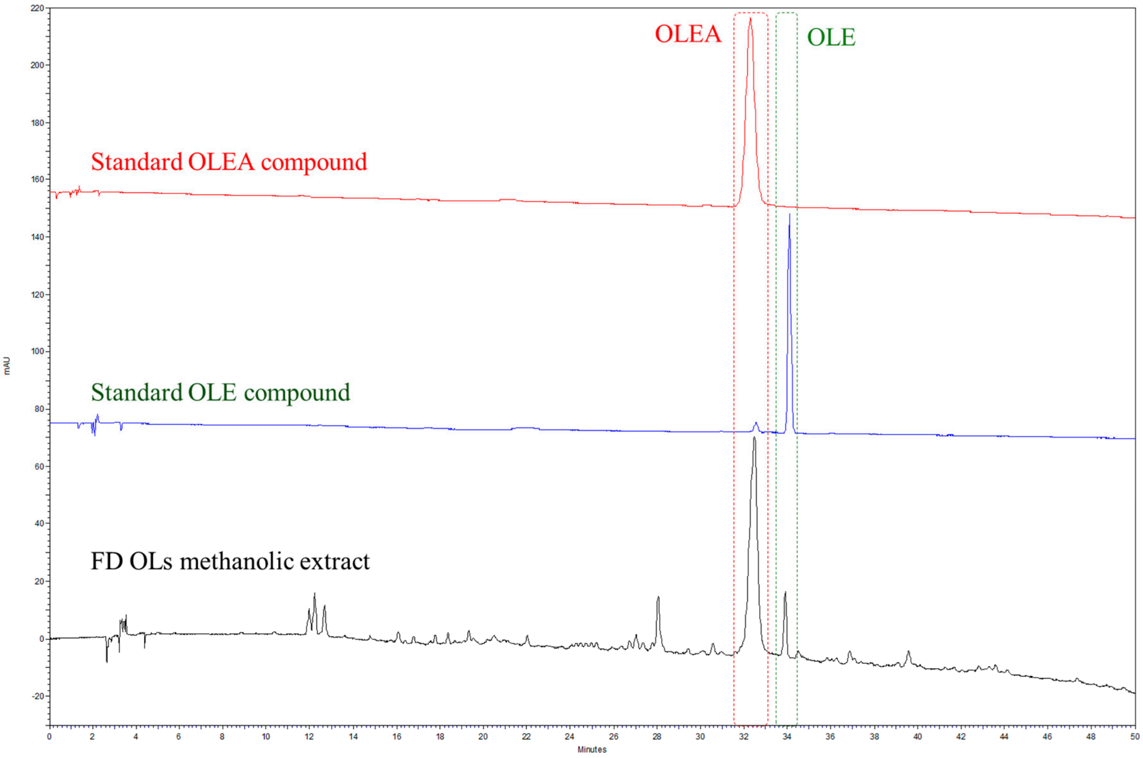

Figure S1. HPLC-DAD chromatograms (at 280 nm) of the methanolic extracts of FD OLs overlayed with the standard compounds of OLE and OLEA. The highlighted peaks are representative of OLE and OLEA.

AA dried OLs methanolic extract

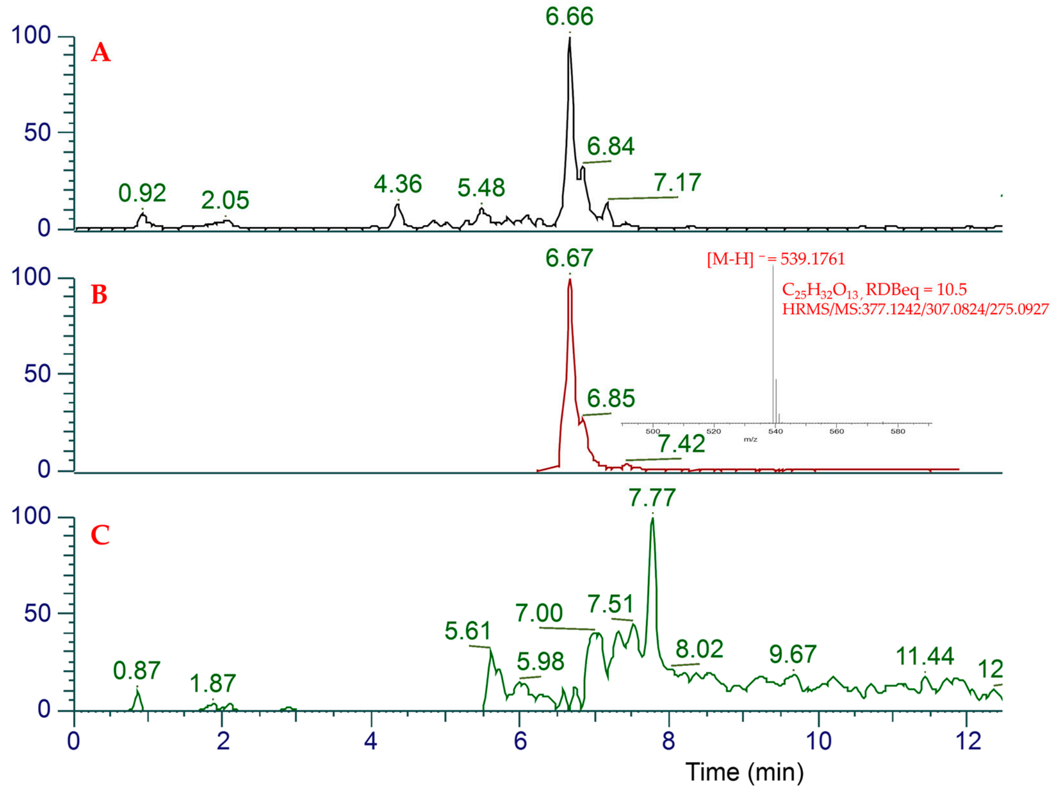

Figure S2. Base Peak Chromatogram obtained by LC-ESI/LTQ-Orbitrap/MS Analysis of the methanolic extract of AA dried OLs (A), Typical Extracted Ion Chromatogram of OLE at m/z 539.1763 (B), Typical Extracted Ion Chromatogram of OLEA at m/z 319.1186 (C)

MW dried OLs methanolic extract

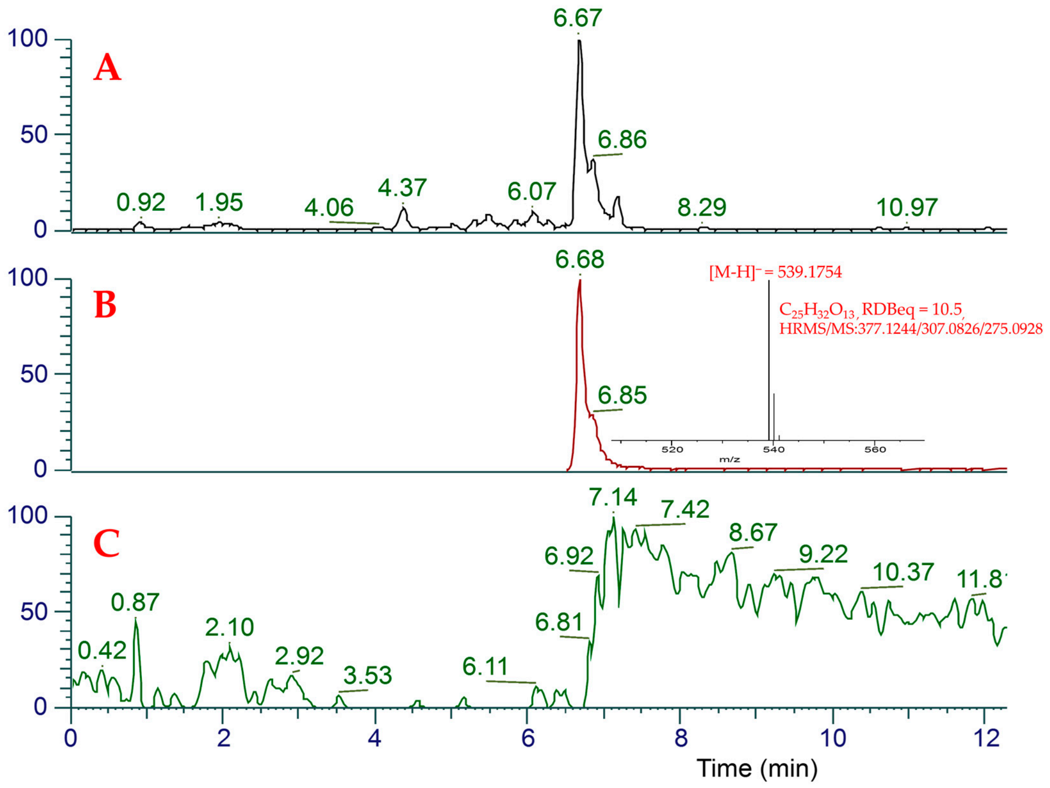

Figure S3. Base Peak Chromatogram obtained by LC-ESI/LTQ-Orbitrap/MS Analysis of the methanolic extract of MW dried OLs (A), Typical Extracted Ion Chromatogram of OLE at m/z 539.1754 (B), Typical Extracted Ion of OLEA at m/z 319.1186 (C)

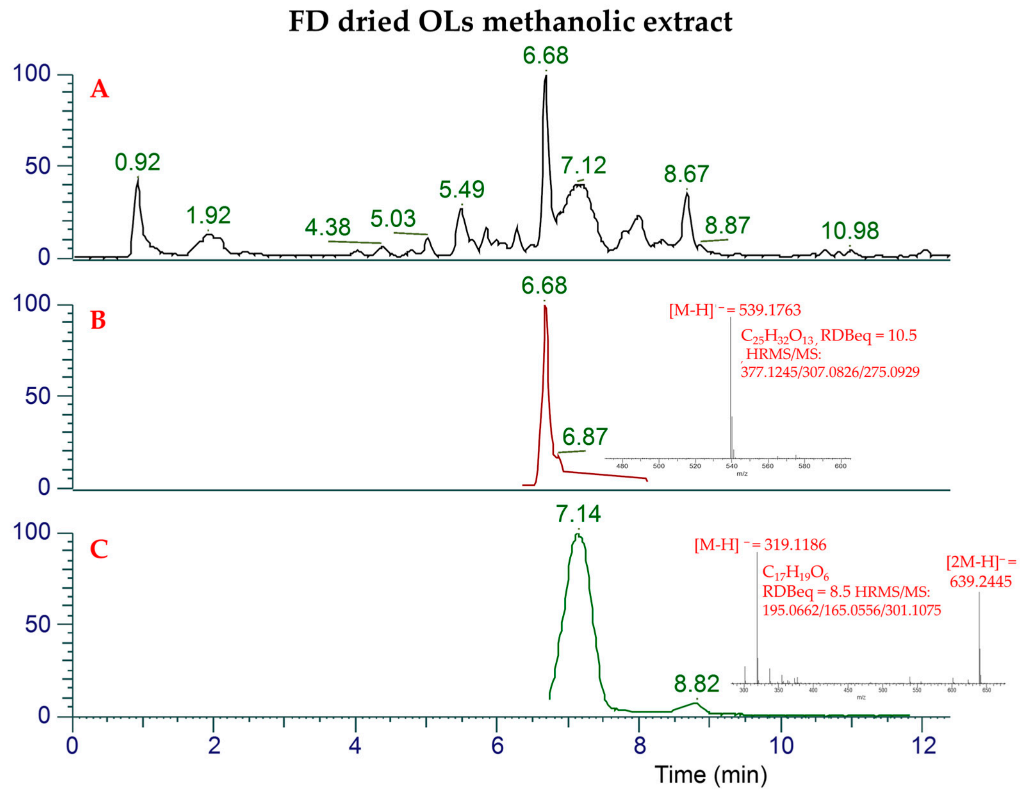

Figure S4. Base Peak Chromatogram obtained by LC-ESI/LTQ-Orbitrap/MS Analysis of the methanolic extract of FD dried OLs (A), Typical Extracted Ion Chromatogram of OLE at m/z 539.1763 (B), Typical Extracted Ion Chromatogram of OLEA at m/z 319.1186 (C)

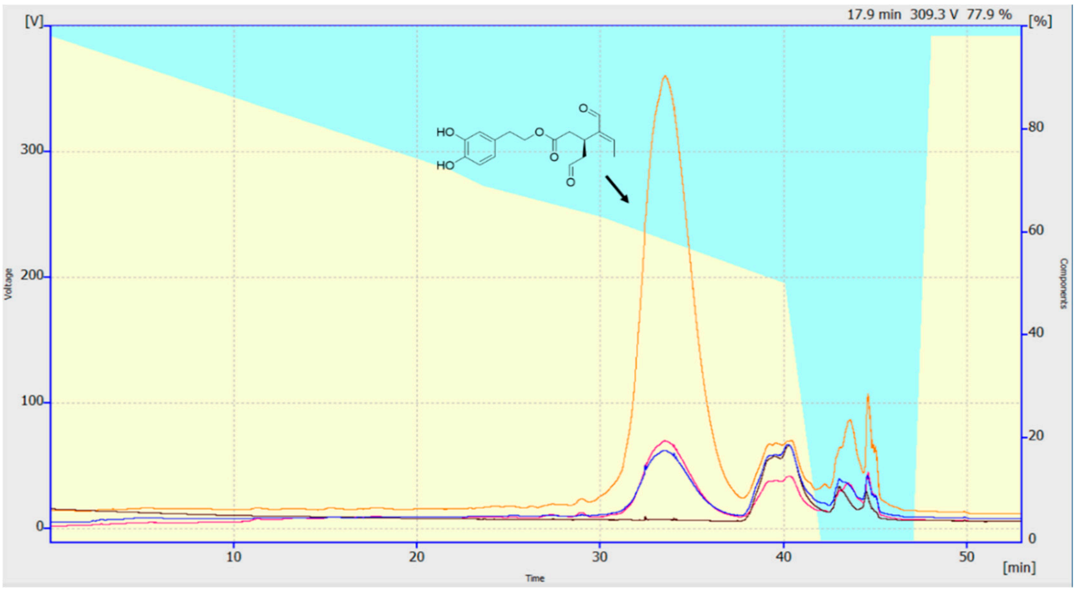

Figure S5. Preparative HPLC-DAD Chromatogram after liquid-liquid extraction with EtOAc of the methanolic extract of FD OLs, with the OLEA peak eluting at rt = 34 minutes highlighted.

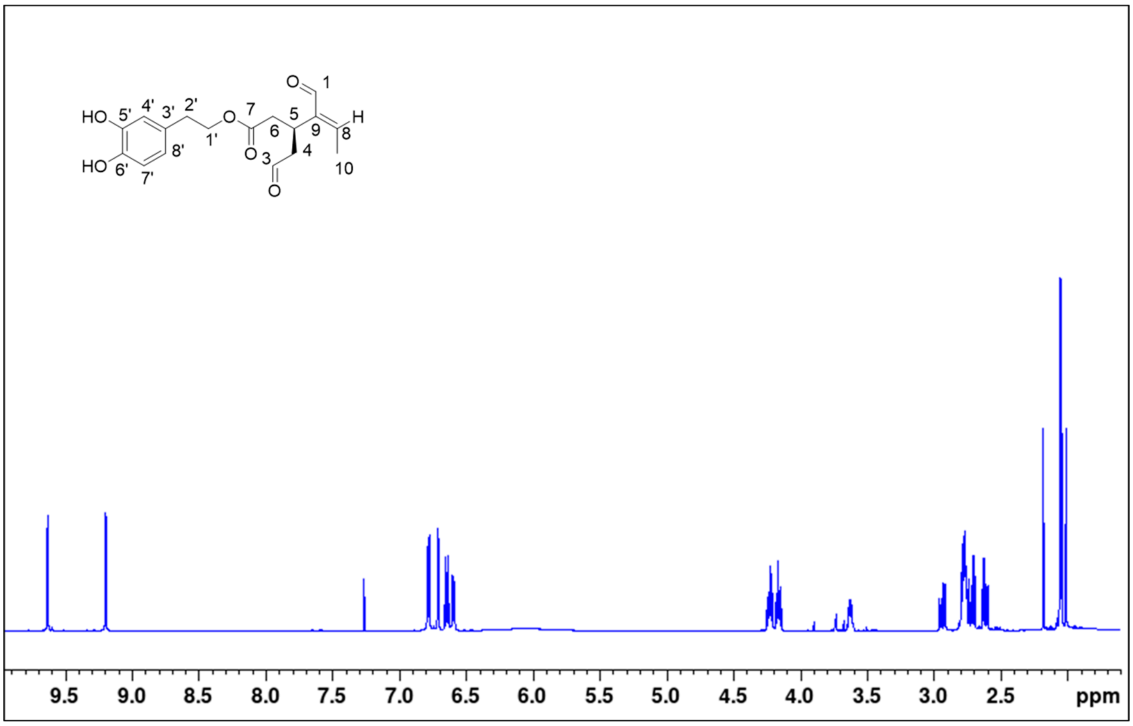

Figure S6. ^{1}H NMR spectrum of oleacein in $CDCl_3$ (600 MHz).

^{1}H NMR (600 MHz, $CDCl_3$) δ 9.63 (brs, 1H, H-3), 9.19 (d, *J* = 1.78 Hz, 1H, H-1), 6.77 (d, *J* = 8.01 Hz, 1H, H-7′), 6.70 (d, *J* = 1.72 Hz, 1H, H-4′), 6.64 (q, *J* = 7.06, 14.12 Hz, 1H, H-8), 6.59 (dd, *J* = 1.76, 8.07 Hz, 1H, H-8′), 4.24 (dt, *J* = 10.89, 6.48 Hz, 1H, H-1a′), 4.16 (dt, *J* = 10.88, 6.29 Hz, 1H, H-1b′), 3.65–3.59 (m, 1H, H-5), 2.93 (ddd, *J* =18.50, 8.23, 1.13 Hz, 1H, H-4a), 2.81–2.73 (m, 1H, H-4b), 2.78 (t, *J* = 6.19 Hz, 2H, H-2′), 2.71 (dd, *J* =15.72, 8.68 Hz, 1H, H-6a), 2.60 (dd, *J* =15.83, 6.46 Hz, 1H, H-6b), 2.04 (d, *J* = 7.09 Hz, 3H, H-10)

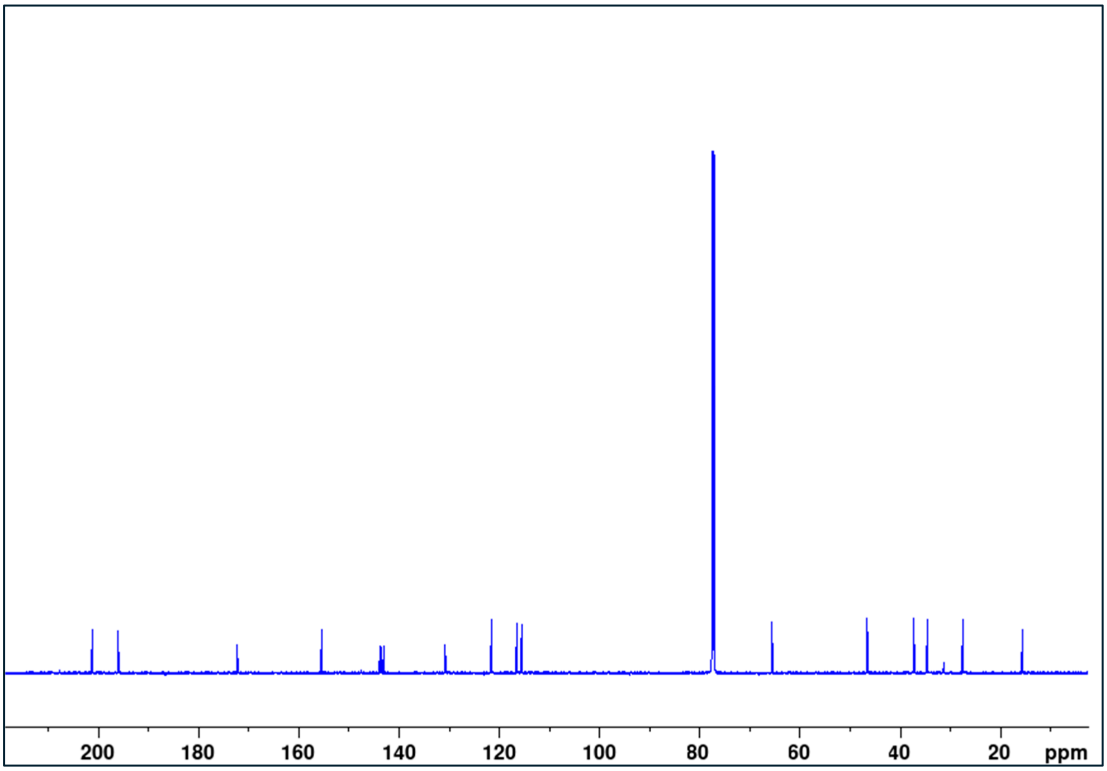

Figure S7. ^{13}C NMR spectrum of oleacein in $CDCl_3$ (151 MHz).

^{13}C NMR (151 MHz, $CDCl_3$) δ 201.08 (C-3), 195.90 (C-1), 172.10 (C-7), 155.31 (C-8), 143.65 (C-5′), 143.29 (C-9), 142.89 (C-6′), 130.59 (C-3′), 121.34 (C-8′), 116.32 (C-4′), 115.38 (C-7′), 65.35 (C-1′), 46.36 (C-4), 37.09 (C-6), 34.37 (C-2′), 27.32 (C-5), 15.42 (C-10)

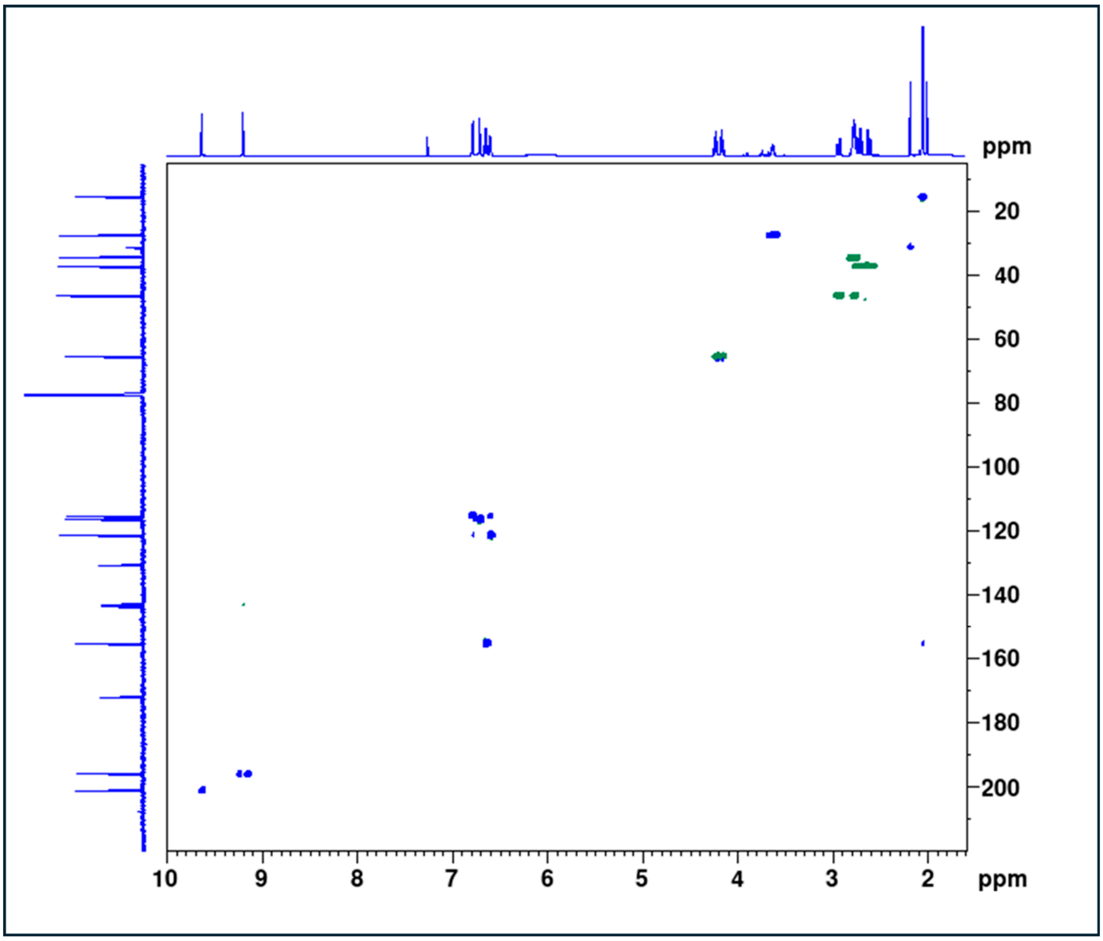

Figure S8. HSQC spectrum of oleacein in $CDCl_3$ (600 MHz).

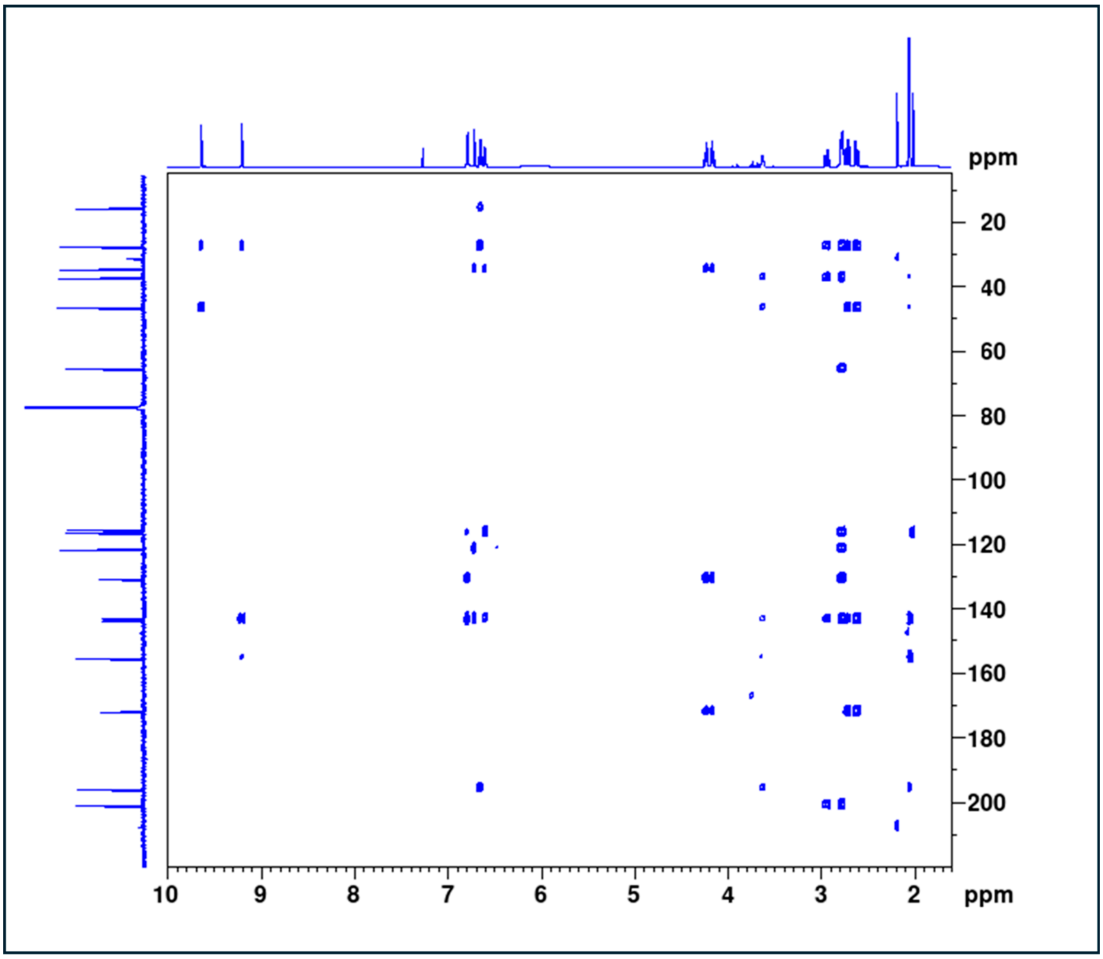

Figure S9. HMBC spectrum of oleacein in $CDCl_3$ (600 MHz).
